# The fertility of moral ambiguity in precision medicine

**DOI:** 10.1007/s11019-023-10160-0

**Published:** 2023-06-06

**Authors:** Jeanette Bresson Ladegaard Knox, Mette Nordahl Svendsen

**Affiliations:** 1 Department of Public Health, University of Copenhagen, Øster Farimagsgade 5, Building, 1014 Copenhagen, Denmark; 2 University Hospital of Copenhagen, Copenhagen, Denmark

**Keywords:** Moral ambiguity, Dilemma, Ethics laboratory, Vulnerability, Justice, Integrity

## Abstract

Although precision medicine cuts across a large spectrum of professions, interdisciplinary and cross-sectorial moral deliberation has yet to be widely enacted, let alone formalized in this field. In a recent research project on precision medicine, we designed a dialogical forum (i.e. ‘the Ethics Laboratory’) giving interdisciplinary and cross-sectorial stakeholders an opportunity to discuss their moral conundrums in concert. We organized and carried out four Ethics Laboratories. In this article, we use Simone de Beauvoir’s concept of *moral ambiguity* as a lens to frame the participants’ experience with fluid moral boundaries. By framing our approach through this concept we are able to elucidate irremediable moral issues that are collectively underexplored in the practice of precision medicine. Moral ambiguity accentuates an open and free space where different types of perspectives converge and can inform each other. Based on our study, we identified two dilemmas, or thematic interfaces, in the interdisciplinary moral deliberations which unfolded in the Ethics Laboratories: (1) the dilemma between the individual and the collective good; and (2) the dilemma between care and choice. Through our investigation of these dilemmas, we show how Beauvoir’s concept of moral ambiquity not only serves as a fertile catalyst for greater moral awareness but, furthermore, how the concept can become an indispensable part of the practices of and the discourse about precision medicine.

## Introduction

New technological and molecular advances aim at transforming medicine into precision medicine. Molecular pharmacology, biomedical research and big data analytics unite in the task of improving disease prevention, diagnostics, and targeted therapeutics by correlating biomarkers and genetic profiles with an individual’s disease history. By way of these advances, the hope is that the right dose, at the right time for the right person can inform precise diagnosis, prognosis and therapy (Sadée and Dai 2005; Schaefer et al. 2020). In accomplishing the goal of early health detection and targeted treatment, precision medicine integrates clinical, environmental, molecular and big data from a variety of sites (Pitini et al. 2020). Concurrently, policy-makers and health lawyers debate what constitutes a robust infrastructure to store and protect vast amounts of personal data generated by new sequencing technologies (Ó Cathaoir et al. 2022). In this way, precision medicine harnesses the expertise of a wide range of professions. Though the disciplines, sectors and institutions of precision medicine border on each other, they are connected by a web of crossroads feeding into each others’ fields of expertise. At the forefront of precision medicine, this close and necessary collaboration is exemplified in the clinical trials of precision oncology. Molecular profiling of a patient tumor tissue is produced in a genomic sequenzing facility and results subsequently circulate back to the clinic where the patient is being treated to help determine patient suitability for the trial and treatment choices. With this set-up, the genomic data generated from this individual patient’s tumor tissue now enters into a data-ecology to generate both health and wealth (Hillersdal and Svendsen 2022; Lee 2021; Tarkkala et al. 2019; Cambrosio et al. 2018). The translational journey between the sequenzing facility and clinic illustrates how specialities are interconnected and mutually dependent. The mix of expertise from diverse sites intersects to provide the best basis for action.

Thus, as the health sciences move from a reactive medical practice based on signs and symptoms of patients to a stratisfied medical practice that draws on medical informatics, biomarker medicine and epigenetics, collaborations across disciplines, sectors and institutions become increasingly pressing. However, the exchange and sharing of expertise between the stakeholders of precision medicine also bring along multiple perspectives on moral questions and concerns partaken to their respective field. Contrary to the term ‘*precision* medicine’, the moral aspect of this interdisciplinary field is far from precise and clear-cut. In this article, we set out to investigate how the moral perspectives are experienced and how they can be discussed across the disciplines in ways which can help inform the work of professionals acting within the field of precision medicine.

While moral ambiguities add suspense to characters in a fictional drama, they create doubts and dilemmas in real-life professions that lack moral absolutes. The stakeholders with whom we collaborated in our research project, *Personalized Medicine in the Welfare State (*MeInWe*)*,^1^ found themselves in situations where moral clarity was often absent either due to conflicting value systems, differing understandings of ethical principles or the novelty of a trial, technique or treatment. Integral to our research project was the propostion to develop a model for the mutually exploratory investigation of moral concerns observed by our research team in their ethnographic sites and expressed by our collaborators in precision medicine. We call the model we designed *the Ethics Laboratory*. The aim of the Ethics Laboratory was to experiment with a horizontal form of collaboration between stakeholders from a variety of professions, ranging from clinicians, biostateticians, epidemiologists to policy makers and social scientists as a way of enhancing communication between the health and social sciences, political institutions and other stakeholders involved in precision medicine. The Ethics Laboratory aspired participants to verbalize, share and reflect on moral issues that surfaced in their work. The overall purpose was to explore these issues as openly, probingly and non-judgmentally as possible (Knox 2021; Knox and Svendsen 2022).

Each of our four Ethics Labs revealed a plethora of moral predicaments, concerns and questions, including conjecturing the impact on Danish society and healthcare decision-making when pharmaceutical companies influence clinic and research through their grants, reflecting on how to create greater transparency in terms of national regulation and translating data from whole genome sequencing, speculating on how to address health disparity when criteria are fluid in the patient selection process and wondering what would happen to the protection of data despite an infrastructure if the collective was run by corrupt politicians. However, our interdisciplinary deliberations demonstrated the omnipresence, at times overtly, other times covertly, of professionals’ unease with the moral uncertainties and implications of precision health. This unease made us frame the questions: Which manifestations or variations of moral ambiguity characterized the four Ethics Laboratories? Which moral themes cut in and across disciplines, sectors and institutions? In analyzing our ethnographic material, we identified two dilemmas, or thematic interfaces, in and across the Ethics Laboratories: (1) the dilemma between the individual and the collective good; and (2) the dilemma between care and choice. As the two themes were frequently repeated among the participants across the labs, we viewed them as presenting two different modalities of moral ambiguity. The article traces and explores these two dilemmas.

## Moral ambiguity in precision medicine

Much has been written on the ethical questions raised by precision medicine. Research, appearing in journals on bioethics and social science, have contributed to both theoretical and empirical insigths, covering a wide range of moral topics, including the ones that dominated our Ethics Labs (e.g. informed consent, justice and care practices). Scholars have pointed out how patient/research participant anonymity could be breached as a result of the linking of sequencing data in biobanks (Kasperbauer et al. 2018; Wjst 2010). Connected to this moral theme is making the principle and practice of informed consent exceedingly difficult to uphold due to the copious amount of complex genomic information about a patient’s disease (Parens 2015). While some suggest ways of improving consent forms as they increase in length and complextity (Glaser et al. 2020), others rethink the understanding requirement in informed consent by distinguishing it from the disclosure of data (Millum and Bromwich 2021).

Other studies reveal a discrepancy between how informed consent appears on paper and how it is translated into practice. Based on anthrolopological research, Hoeyer and Hogle call for studying informed consent as a practical problem rather than an ideal that does not necessarily agree with stakeholders’ “living conditions, the socioeconomic and political realities through which consent is produced” (Hoeyer and Hogle 2014, p. 357). Sun and Ching point to expressed concerns by clinicians and biomedical researchers in Singapore, Canada and the United States about significant limitations in how precision medicine can serve public health purposes, arguing against a troubling, yet still prevalent view of medicine that “divorces disease from social structure and culture” (Sun and Ching 2021, p. 458) with consequences for social equity and fair accessibility to care.

Critical of the notion of human atomistic autonomy, Prainsack (2018) builds on the concept of relational autonomy seeing that decisions made by patients are largely shaped by their relationships to others (e.g. family members, friends, clinicians, etc.). This realization of the interdependence of people directly impacts how precision medicine is enacted, according to Prainsack, as it opens up for the practice of solidarity that combines self-interest and other-interest, or personal and collective needs. Similarly, these social-economic-political determinants of health plays a defining role in Lee’s recent research that discusses the concept of responsibility within the framing of gift-giving by offering one’s biospecimen or electronic health data to precision medicine research (Lee 2021). Defending a relational ethics entrenched in social solidarity, Lee argues for the inclusion of reciprocal responsibility as a way to address issues of justice and equity in health care, such as lack of access and affordability and uneven representation in genomic datasets. Achieving justice calls for a demonstration of benefits for “the most vulnerable groups” (Lee 2021, p. 61) who are asked to participate in precision medicine research, whereby she extends the giver/participant-receiver/researcher relationship to more than an exchange of genetic data to encompass a concern for participants and their communities.

Viewing this scholarship on the ethical aspects of precision medicine from a broader perspective, it seems to be characterized by two primary, mutually isolated approaches. One approach focuses on describing its challenges and to suggest solutions to problems or recommendations for improvement. This form of approach in the literature usually calls for a context-dependent approach to emerging precision technologies or has an interventional aim (i.e. the creation of special informed consent forms in relation to whole genome sequencing). Another approach in the literature typically relies on ethnographic studies using interviews and participatory observations to learn about lived experiences for patients, relatives and/or healthcare professionals and other stakeholders. Our study differs from both forms of approaches. Through an intervention (the execution of four Ethics Labs) we set out to understand stakeholders’ lived experience in order to access a novel engagement with the nature of their moral concerns. In this way, our study contributes empirically to the literature by merging intervention and lived experience. Theoretically our study advances scholarship by interpretating stakeholders’ concerns through the concept of moral ambiguity. Despite the impressive display of moral perspectives in the literature mentioned above, we are not familiar with studies that specifically hone in on the common denominators when diverse professional fields convene to discuss their moral concerns, if they convene at all, or what they might be. In fact, the key lesson drawn from the scholarship mentioned is how perplexing, multi-layered and versatile the ethics of precision medicine is.

Moral concerns arise on sites of precision medicine when professionals are unclear about how best to act or pose questions to the possible ethical implications of a drug, trial, treatment, policy, etc.. Carlo Leget perceives boundaries as a “matter of perspective” (Leget 2006, p. 256) which causes them to fluctuate and take on different appearances, yet boundaries also “embody the chance for communication with what is beyond” (Leget 2006, p. 256) them as a way to elucidate a morally murky terrain. The Ethics Labs emerge, in fact, as a chance for conversations with what lie beyond these boundaries. Through our study of professionals’ lived experience with this murky moral terrain, we wish to depict the complex interweavings, or relationality, of various fields and practices in precision medicine and, in doing that, how they connect, simultaneously, to the moral compass of stakeholders and to larger societal concerns. We treat the moral themes that came up in the Ethics Labs as dynamic border zones between perspectives, concerns and interests. These themes were grounded in everyday experiences of moral ambiguity, thus harboring the possibility for communicating across disciplinary boundaries.

Thinking with Simone de Beauvoir’s notion of *moral ambiguity* (Beauvoir 1948/2018), we understand the term to represent the intricacies and nuances of moral experiences in concrete situations with others (i.e. colleagues, professionals from other fields) or objects (i.e. protocols, instruments, policy papers). With Beauvoir, we can explore the moral predicaments caused by precision medicine not as something extraordinary or rare but, more exactly, common across fields. Moral ambiguity, to Beauvoir, is a condition underlying human existence and stems from the interrelational nature of subjects situated in the world, or, in other words, the constant tension between the internal and external, between consciousness and materiality (Beauvoir 1948/2018, p. 7). As free situated beings, individuals are not dictated or guided by any pre-existing moral law or moral absolutes in terms of determining best action. By illuminating actual moral experiences without positioning them within universal or prescriptive categories, we can expose the concerns, predicaments and questions posed by professionals within precision medicine, or in other words, the moral ambiguity of precision medicine. Framing this study within Beauvoir’s concept accentuates the porous boundaries between right and wrong that emerged in the Ethics Labs and, thus, the moral border activity in which the participants were involved. Moral ambiguity amplifies the pressing issue of how, where, why and when to set boundaries in morally complex situations.

Sartre and others (notably Camus, Marcel and Merleau-Ponty) perceive ‘situation’ as an ontological term, positioning the human being in the world and as an embodied reality. ‘Situation’ refers to the factual conditions in which the individual lives. A human being cannot *not* be in a situation. It is an ontological fact. One single situation at a time. Beauvoir adds a specific moral interpretation to ‘situation’ as her notion encompasses a moral concern within a situation. Moral choices impact the world, including the people around us (the immediate sphere either professional or private) or people we may not even see (the social or societal sphere). She inserts a moral responsibility that is directed toward the other in a given situation. In her interpretation, Beauvoir’s notion of ‘situation’ can inform the tension and negotiation in the two dilemmas that our study identified. At the heart of any situation lies the potentiality of harming ‘the other’ either taken in its singular form (e.g. patient) or as a group (e.g. society). Taking ‘other’ both in its singular form and in its collective form, it was clear that most participants were riddled by this potentiality. By understanding moral ambiguity within a notion of ‘situation’, a moral decision-making process can suspend pre-established, fixed principles to allow for reflections on the particularities of lived experience. We are not discounting ethical theories or bioethical principles. Rather, we are questioning the approach to using them in practice. The Ethics Laboratory functions like a lab that is driven by curiosity with the clear intent not to end an inquisitive and open deliberation in its infancy due to overruling principles. Prescriptive ethical frameworks tend to dissolve ambiguity and neglect the fluctuations and variations of concrete situations. Though consideration to moral duties and consequences serve the purpose of providing action guidance, an unwavering and dogmatic attention toward them can prematurely disregard the moral murkiness of situations. The framework of moral ambiguity serves to advance a deliberation on the moral complexity of a practice. Moreover, it allows for moral discomfort through which participants can share their vulnerabilities. In the Ethics Laboratories, we explore selected themes (i.e. selection, data, interpretation and private–public relationships) without putting specific ethical principles in participants’ mouths before or during our deliberation. In other words, moral ambiguity creates a fresh mindset, a meditative pause, that eventually helped us discover manifestations of vulnerability, integrity and justice in precision medicine.

## The empirical setting

Between December 2019 and September 2020, first and second author hosted four Ethics Laboratories at the Department of Public Health at the University of Copenhagen. One lab took place in December 2019, one in February of 2020. The last two labs were scheduled to take place in March and April of 2020 but were eventually delayed by 5 months due to the Covid-19 pandemic. As the Ethics Laboratory depends on active onsite participation, they could not be moved online. Participants held high-ranking positions that left little time for participation in extra professional meetings, let alone a 3 h workshop based on an experimental concept. As part of the MeInWe project, 10 ethnographers had conducted participant observations and interviews in laboratory and clinical sites, and among patients and policymakers, to study the introduction of precision medicine in Denmark. Participants in the Ethics Labs were collaborators from the ethnographic sites. Undoubtedly, access to participants was enabled by the MeInWe researchers’ already well-established collaboration with the fields from which they came.

Prior to the actual Ethics Laboratories, first author set up preliminary meetings with members of the MeInWe research group to collectively reflect on two topics: one was to lists candidates who had the opportunity to participate despite the Covid-19 pandemic and who had alluded to or shared their ethical challenges in working in precision medicine; and the other was to consider some of the key ethical themes that their fieldwork had revealed. Among an assortment of recurring themes, the research team and PI identified four major themes for four different Ethics Laboratories: selection, data, interpretation and private–public partnerships. Project collaborators were invited and Ethics Labs were carried out with first author as facilitator. All four Ethics Labs followed a dialogical structure that centers around two exercises: starting out with one where participants (our own researchers as well as our collaborators) reflect on actual work encounters with the lab theme and choosing one example. This exercise is followed by a second one where they reflect together with a partner, standing in front of large post-its on the wall that describe their individual example. Subsequently, participants share their findings with all lab members.

All Ethics Labs were audio recorded and subsequently transcribed verbatim. In total, 20 participants came from outside the MeInWe group (external participants) and 11 participants came from inside the group (internal participants). Professionally, the Ethics Laboratories were represented by expertise in qualitative research, ethical practices, genetically rare diseases, precision oncology, cardiology, diabetes, biostatistics, policy-making and health law. Following the execution of the four Ethics Laboratories, first author interviewed 8 participants, four from outside of our research group and four from inside our research group (see Knox and Svendsen 2022). Due to the Covid-19 pandemic these interviews were conducted on Zoom. They were subsequently recorded and transcribed. Both authors had several informal conversations with participants during the breaks in each of the four labs.

## Justice matters: the dilemma between the individual good and the common good

Beauvoir explains in *The Ethics of Ambiguity* (1948) how ambiguity is firmly constructed in a phenomenology of moral experience and how this experience particularly manifests in the interpersonal and social aspects of being an individual. In the Ethics Labs, we observed this interpersonal and social aspect in the form of an awareness of being a professional within a collective. The Ethics Laboratories quickly revealed how participants reflected on the societal impact of precision medicine and not just their own practice. They seemed to move within a fluid boundary between their field of expertise and society, revealing hybrid identities: that of a professional within precision medicine and that of a conscientious citizen in the Danish welfare state. This dilemma expressed participants’ unresolved state between, at times, conflicting concerns, values and responsibilities.

Ambiguous situations beget vulnerability. Vulnerability reaches beyond the mere existential frailty and human condition of the individual as it is often portrayed in the phenomenological-existential tradition (Butler 2016). The vulnerability that the first dilemma exposed connected to relationality or embodied relations within a public health context. Vulnerability is thus understood within a larger infrastructure of interdependent relations, e.g. being caught between individul care practice and public health interests, transforming vulnerability to a socially induced condition. Naturally, interdependent relations make for entanglements and consequently questioning, when caught up in them, or framed within our context: how am I to consider a moral situation that thrusts me between ‘I, the professional’ thinking of obligations to my profession or this individual patient/research subject and ‘I, the citizen’ thinking of the collective good? This vulnerable—and complex—interface between the individual and the collective good emerged already in our first lab on selection.

Selection is essential to the development of precision medicine and is performed in multiple places, such as in the clinic, in laboratories, and at the governmental level. The Ethics Laboratory on selection consisted largely of physicians working with experimental oncology in phase-1 trials, monogenic hereditary heart diseases, and rare genetic diseases in children. Sarah is a precision oncologist in a phase-1 unit. In her unit, they test drugs that have previously only been tested on animals. Their patients have exhausted all other forms of standard cancer treatment and have been selected as suitable for the clinical trial. Once selected, the patient undergoes a whole exome sequencing of tumor tissue to identify the mutation that drives the formation and growth of their cancer (i.e. the ‘driver’). Presenting her case in the Ethics Lab, Sarah starts out by posing a general, yet potent question “Should genome sequencing be for the very few or for all of us?” It forcefully captures her case. Without wording it as such, her concern touches on the legitimacy of her clinical work in the collective. She explains, for example, how her unit looked at the number of biopsies that led them to ‘drivers’ upon which they can target a treatment. The number was around 20%. However, not all 20% will necessarily benefit from the targeted treatment. In fact, “based on this huge group of patients who get a biopsy [with the chance of getting targeted treatment] less than 5% are eligible.” Sarah finds herself torn because targeted treatment is succesful for a minute number of patients, reflecting on precision medicine being for the few rather than for ‘all of us’,^2^ though it is politically packaged as if it is. The exclusive early phase clinical trials make her wonder: “Is it fair as a society to spend these resources on the very few?” and yet “if we do *not* offer it to all, then it is definitely something that will increase inequality in health care and in cancer treatment.”

Being stretched between her professional commitment to do good for the patient in front of her and her societal concern for the fairness in helping only a few, Sarah reflects further on her predicatement when she adds yet another moral nuance, namely determining *who* among her patients are suitable for treatment (Dam et al. 2022). Commentators have expanded Sarah’s moral discomfort to adjacent moral challenges such as the selection of proper inclusion criteria to avoid undersampling of minorities (Popejoy et al. 2018; MacKay and Saylor 2020) which could, if not done, lead to a widening of the health care disparity gap. Precision medicine research is predominantly based on populations of European ancestry, making its equitable diffusion into clinical care difficult (Landry et al. 2018). The societal concerns of collaborators who participated in the four Ethics Labs mirror a growing trend in scholarship about the collective good, fair access to precision health services and health costs (Prainsack 2018). Departing from a specific position of individual rights, Morrisey and Walker (2018) emphasize in their study the imperative to include a social justice perspective, or community rights, on population preventive genomic sequencing to prevent unequal access to precision health. Recently, Jenny Reardon argued how bioethics should prioritize the concept of justice to ensure that society’s moral vision for genomic medicine rests on a collective, inclusive reflection and social responsibility (Reardon 2020). Reardon’s article appeared in a special issue of the prestigious Hastings Center Report. In this issue, editor Joel Michael Reynolds encapsulates medicine’s chief virtue when he states that “justice is medicine’s lifeblood, that by which it contributes to life as opposed to diminishing it” (Reynolds 2020, p. S3). In the Nordic countries, precision medicine is embedded in larger structures that make up society as a whole where communitarian ideas of the common good are framed by the state (Jensen and Svendsen 2022). The first Danish strategy for precision medicine explicitly states that precision medicine is intended for the benefit of patients (Ministry of Health and Danish Regions 2016). Though this is a noble strategy, it raises ethical questions as demonstrated both in our Ethics Labs and in the literature.

Additionally, several participants wondered whether the intentional good of precision medicine is drowning in a regulatory soup (Nicol et al. 2016) and viewed the situation as potentially preventing maximizing overall welfare. One of the geneticists, Evelyn, working in a clinic for rare, genetic diseases portrays a possible harmful outcome of the societal protection of individual data. Evelyn explains how her clinic is prohibited from recontacting a patient a year after completion of a treatment even if new knowledge of the disease has emerged or new knowledge has emerged about the risk of a specific complication. Evelyn mimics several studies that discuss the moral obligation to inform research participants of the reinterpreted results of their initial genomic analysis (Appelbaum et al. 2020; Otten et al. 2015). The situation she and these studies depict creates an unsettling paradox where the societal task of protecting individual data can end up hurting individuals. To these participants, it is a paradox that does not seem to promote fairness or a sense of protecting the common good. 

In the Ethics Lab on private–public partnerships, Simon (a geneticist) expresses a different version of responsibility towards society. He explained a sense of great unease with how to interpret the regulation in regards to biobanks. The law implies that he must constantly consider the individual's involvement and data protection, but this means that he can be caught in situations where he cannot help the individual citizen/patient. It made for an unsafe situation that effectively could harm citizens/patients: “I get worried because I want to act correctly in the eyes of society and I don’t want to violate patients’ interests. I want to follow the rules laid down by society […] but I don’t understand them and I worry that one day I will do something illegal or wrong without knowing it.” The lack of transparency in the law made it difficult for him to assess fair action. Opaque regulations do not serve health providers or citizens/patients because of the range of interpretation. If he ignores the legal regulations, he will improve public health, but in return he risks not respecting individual privacy. Others across the four Ethics Labs echoed his sentiment and found the predicament increasingly frustrating. The predicament highlighted the potential discrepancy between serving the individual good and the collective good. 

Simon also questioned whether an agreement between private industry, the university and himself sat well with his moral beliefs. The collaborating (start-up) firm had an altruistic angle on drug development as it wanted drug production to be more democratic and serve the principle of universal healthcare. The basic idea was to pool a hundred or so small investors to finance the development of a drug distributing Intellectual Property (IP) among these investors. Simon explains the ethically sound aspect of the firm’s mission when he states that “the firm wanted big pharma not to keep all the money to themselves but to have others [i.e. users of a drug], for examples diabetics get co-ownership of the insulin they inject into their thigh”. As it was a daunting task for his university to make individual contracts with numerous small investors, the solution was to drop the IP rights and make a contract research agreement. This meant that the firm would pay the university to develop the drug and the firm would receive the profit from selling the product. The firm insisted on Simon getting a share of the profits, i.e. getting IP rights, so that he would become co-owner of the product where it would usually be the university having IP rights. The moral issue, as he and others in the Ethics Lab pointed out, was the possible moral dilemmas that the agreement put him in. For example, working on several other research projects, he may be inclined to devote more time on the project where he has a personal financial interest. Simon wrestled with his convictions about where to set the boundary for the right involvement in an endeavor that was initially to serve democratic purposes and, thus, how far he was willing to take a collaboration and avoid financial improprieties. The predicament illustrates an embedded conflict in the health care industry between self-interest (e.g. monetary return) and a self-less service for the promotion of human health. On a different scale, Reardon poses in her article similar concerns when she writes “should a mode of doing research so dependent on speed, technological innovation, and venture capital dominate the life sciences?” (Readon 2020, p. S72). The entanglement of genomic medicine and venture capital makes the researcher vulnerable for primarily serving his/her personal and/or professional interests, jeopardizing the sharing of knowledge due to competition and the moral fiber of the researcher. As a consequence, it would make it more difficult for the researcher to become a conscientious objector to a contract, practice or an action.

During first author’s interviews after the four Ethics Labs several informants from a variety of fields made the same observation of the moral rivalry between the individual good and the common good (see Knox and Svendsen 2022). Katherine, who is a member of our research team, remarked on the schism that she was fascinated to see “how ethically reflective they [participants from outside the research team] were in their different fields of expertise, not only in regards to their own field but, particularly, in regards to society, like asking about where the health care system is going and what ethical challenges precision medicine confronts society with.” The balancing act between thoughts on the distribution of goods and determining the right moral conduct in a situation expresses how they understand themselves not only as professionals but as agents who impact the fair distribution of health in society. They extend their sense of professional responsibility to encompass a societal responsibility as their medical decisions reverberates outside the professional setting within which they work. In this sense they demonstrate a solidarity towards larger collectives beyond the medical world (Prainsack 2018; Lee 2021). They seem to harbor a responsibility for foreseen, i.e. high risk factors or predicatable results, *and* unpredictable consequences of inequality, i.e. imaginary outcomes that they fear can or will happen but has yet to manifest. This responsibility that lies in a moral border zone plays a role in their interpersonal interactions and appeared important for self-perception. It mattered to them that their profession be morally alert to potential consequences for the individual patient or larger patient groups and the collective. They are acutely aware of their profession engendering serious ethical questions of justice and equal access to healthcare, and they posed questions about how to address the clash between helping a few patients when the vision of precision medicine is to help all of us and how agents of precision medicine is to promote their commitment to fairness under the current circumstances. In various ways, participants thus expressed how social responsibility includes consideration for societal management and deliverance of health care.

## Integrity matters: the dilemma between care and choice

Presenting a patient or research subject with choices is well meaning and tied to the notion of autonomy. The intent is to empower an individual and secure his/her judgment abilities. The notion of autonomy, however, also risks desocializing human agency (Martin 2013). Though not specifically aimed at precision medicine, Annemarie Mol describes how the ideal of presenting patients with choices (a new drug, a new trial, a new experiment, etc.) can be at odds with good care as many people are burdened by choices (and thus at risk of not making good ones) that involve uncertain futures, such as in the case of serious illness (Mol 2008). The notion of choice promotes the individual as an active, responsible participant in healthy living. Mol encourages a rethinking of such a logic of choice considering that the complexity of healthy living cannot be reduced to a simple matter of choice. She suggests a suitable alternative in the logic of care that points to the socio-cultural, normative context within which a choice is carried out and where individuals’ bodily fragilities exist. She reminds us that good care grows out of collaborative attempts to attune the advancements of knowledge with the needs and life story of patients (Mol 2008). Caring, embodied relations demand attuned attentiveness and point beyond the rationalist version of the human that lies within the logic of choice. Though Mol talks primilarly about patients, any stakeholder in the healthcare system can be entangled in these logics.

Across the Ethics Labs participants identified moral conflicts that seemed to fluctuate between choice (i.e. right to be informed and make autonomous choices) and care (i.e. virtues that strengthen attention, connection and caution). In this way, precision medicine can be perceived as stretched between caring practices and the creation of possibilities for health prevention and improved treatment. A conflict can arise, as discussed in one lab, when pressing policies and/or guidelines intend to generate knowledge that in the future will pave the way for new treatments and, thus, facilitate options for patients. However, the guidelines can deter the impulse and imperative to care in the present moment. The tension between care and choice can also arise under the cloud of the uncertainties in precision medicine. These uncertainties made many participants in the Ethics Labs, both physicians and non-physicians, reflect on how the field may impact the physician–patient relationship. 

In the Ethics Lab on selection, Sarah grappled with the many uncertainties of genetic profiling in experimental cancer treatments. Sarah has found herself in situations where she, despite doubts, made the decision not to include a patient in the clinical trial treatment. Her doubts consisted of care and concern for the patient: is she/he strong enough to withstand the treatment yet sick enough to qualify for treatment and can a patient’s autonomy be ensured if the health data she/he has to consent to is, de facto, incomprehensible to her/him? Like the logic of choice, precision medicine promulgates options and the freedom to choose between them. However, choice can also be deceitful when, for example, an individual—be it a patient, research subject, clinician or researcher—struggles to understand the information given or when test results reveal a plethora of genetic unknowns. Delivering dense medical information that leaves the recipient perplexed and perturbed can cause moral concern in a clinical and/or research setting and compromise informed consent as a foundational principle in practice (Hoeyer and Hogle 2014).

In our Ethics Labs, the tension between Mol’s two logics seemed intertwined with another foundational value in research and the clinic, namely integrity. An unwavering ambition for a logic of choice that the national and global push for precision medicine seems to promote can dwarf the integrity of care and trust relationships that thrive on process and ethos whether it be within clinics, laboratories or governmental institutions. Integrity is, not surprisingly, much lauded in healthcare (Edgar and Pattison 2011). In a way, integrity has become a bedrock virtue for caring practices. In fact, it is deemed imperative for professional flourishing as it expresses fidelity to moral character, conscience and an integrated set of moral convictions (Beauchamp and Childress 2001; Williams 1981). However, integrity can be compromized when professionals cannot perform in accordance with core values of care that the logic of choice undercuts. Seing that this logic is foregrounded in healthcare, it can be a morally consequential challenge to contest it. This creates a situation that makes healthcare agents vulnerable. In this way, integrity has become entwined with vulnerability, a concept we traditionally only attribute to research subjects or patients within a health care setting (Zaner 2000; Mergen and Akpinar 2021). Physicians within healthcare rarely communicate their vulnerability and doubt (Sample 2011; Gulbrandsen 2018), still we observed the presence of both in the Ethics Labs, not only among the participants who are physicians but among other professionals. Unlike Butler’s reframing of vulnerability within a social or even societal context (Butler 2016), the vulnerability in the second dilemma had a distinct internal character while still being relational in nature. In addition to being concerned with the ethical dilemma that could arise between a specific good for an individual and societal good as a whole (dilemma 1), participants described burdensome situations where acting in accordance with professional and/or personal integrity was challenged, potentially undercutting moral governance. During the Ethics Labs many participants reported that they felt unprepared to deal with the ethical morass of precision medicine within their own field of expertise. By taking into account the moral ambiguity of the human condition (Beauvoir 1948/2018), we can understand the professionals as expressing how its inescapable tension can be uncomfortable, yet imperative to navigate in.

As stated above, the uncomfortable moral struggle participants experienced was internal, yet relational. This dualism emerged when, for example, Sofie (a social scientist from our research group) recounted how she had interviewed a clinician in a clinical research unit, who expressed the moral discomfort of inviting patients to participate in a protocol while knowing that his unit has an economic interest in enrolling patients. The clinic and the offered treatments rely on enrolled patients creating a situation where it becomes dependent on some industrial and commercial interests in order to provide treatment. Developing new technology and treatment, pharma companies work from a logic of choice that supports an atomistic autonomy (Prainsack 2018) intending to empower individual decision-making. Yet, the clinicians can be caught between having to promote and protect patient autonomy (mandated by the logic of choice) while knowing how central relationality (e.g. patients conferring with loved ones about weighing an unknown future scenario against another) is to patients’ decisions (Mol 2008). The clinician along with the other participants in the Ethics Labs didn’t rely on abstract mandates or theoretical frameworks in moral predicaments such as these. They exercised moral caution; they paused and pondered the fluid boundary between right and wrong action. Entering into a field that is fraught with ambiguity can be exhausting and cause disturbing moral qualms for the individual stakeholder. Being unprepared, as many of them were in their own mind, can compromise a course of action and deepen the moral predicaments.

Tied to vulnerability is a sense of responsibility towards something of value and the impulse to do right by patients. Martha is a precision oncologist working with immune therapy. In her Ethics Lab, she explains her qualms with having to communicate extremely convoluted information retrieved from genetic sequencing in a short consultation that needs to cover diagnosis, treatment plan, side effects, etc. Her qualms were echoed by both physicians and non-physicians. Precision clinicians are called upon to assist patients in understanding the onslaught of genetic information, often with unknown clinical implications, creating uncertain future prospects for patients and their families. Taking her patient through options relevant to her/his case and asking to sign the informed consent forms that exist to prevent legal complaints and being sued for liability, Martha also expresses a concern about these forms. In her mind, it is “a little dishonest to patients” to include certain information in the patient file that gives the patient the impression that they screen for a particular mutation when they don’t. In addition, she believes that the information is so complex that it may only be understood by 10% of them whereas “the rest just say ‘where do I sign’”. The situation made her uncomfortable and weary as for how to resolve it. Premised on a logic of choice that mandates her to explain her patient’s clearly demarcated options, she indirectly expressed how this logic could end up halting a logic of care that is far more equipped to deal with uncertainties and unpredictability (Mol 2008). As precision medicine is heavily data-driven, it may demote a person-centred care where the ill is reduced to a collection of data, and thus raise new ethical issues concerning decision-making. Martha’s situation touched on a neighboring issue that is distinctive to precision medicine: the challenge for clinicians to understand the complexity and volume of genomic data generated by molecular biologists and bioinformaticians. Several participants volunteered their vulnerability as they shared their self-proclaimed inadaquency in, at times, interpreting data and the ethical burden that came with it.

Elizabeth is a chief governmental administrator who works to establish an infrastructure for precision medicine in order to protect the sensitive and vast genomic data of citizens/patients/research participants. She raises yet another novel ethical challenge in precision medicine. Storing, linking and sharing data has created the pressing question of data security. Informed consent forms are part and parcel of the infrastructure of which Elizabeth speaks. The many discussions on the wording of these forms revealed, Elizabeth explains in her Ethics Lab, the need for further training in genetic counseling because it is improbable that healthcare providers can interpret patients’ genomic data. A member of the same Ethics Lab echoed her point while expanding it to say that specific training is needed in other key areas in the healthcare system, explaining how Research Ethics Review Boards lack the qualifications to assess their research protocols. These illustrations of doubt and uncertainty expose a distinct moral dimension to precision medicine and how the volume of data does not necessarily equate with proper care and caution. Participants’ examples spoke of an apprehensive relationship between technology and care with the risk of turning the health care professional into a ‘broker of choice’. The forecast of some commentators is, however, that “precision medicine, for all its futuristic promise, will ultimately depend on some of the oldest medical skills” (Eyal et. al. 2019, p. 815), i.e. being able to communicate and connect with the patient/research participant/relative/citizen/colleagues. Advancements in biotechnology and medical science, as illustrated by precision medicine, is geared to empower the individual autonomy of patients and citizens (Sabatello and Appelbaum 2017), yet they do not automatically imply good care (Mol 2008).

Volunteering one’s individual data for research is often viewed by research participants as a way of joining the collective and contributing to overall welfare even if this collective good is in the future (Olsen et al. 2020). An epidemiologist Martin and a couple of geneticists in the same Ethics Lab explained that legislation, for example on GDPR, can hinder citizens from participating in the collective good because access by researchers to their genomic data is made virtually impossible. Regulation, Martin states, is based on the false notion that individual data in itself is highly useful when, in fact, this data is only meaningful when compared with a voluminous amount of other people’s data. The regulation that intends to promote good can end up preventing good as it can impede researchers from advancing their study and producing results. Martin and many participants in other Ethics Labs shared experiences that illustrated a morally challenging rift between expressions of care in the moment and the push for enabling future choices generated by increasing knowledge of diseases and, thus, improving the treatment of future patients. 

Rendtorff cautions against striving to eliminate vulnerability to create flawless human beings (Rendtorff 2002) without the courage to be uncertain, to question and wrestle with moral boundaries. The participants in the Ethics Labs, though coming from diverse professional backgrounds, were on the same precarious grounds in their work with precision medicine. It made for the symmetry between them one of vulnerability. Vulnerability opens a portal to reflection through which “a coherent integration of aspects of the self [and] being faithful to moral values” (Beauchamp and Childress 2001, p. 36) can be examined. There is an integral relationship between vulnerability, e.g. sharing doubts, qualms, concerns, and integrity in that the latter necessitates the embrace of vulnerability in order to honestly align with shortcomings such as being uncertain about a test result or who to select among patients. Vulnerability and integrity as adjunctive virtues seemed cardinal to the participants’ professional life though neither virtues were explicitly mentioned as something that morally characterized their situation, yet their constant moral questioning and negotiation disclosed that something of immense value was at stake and in danger of being blurred or even breached. Mol’s logic of care teaches agents operating within healthcare to be acutely aware of attuning knowledge (e.g. new drugs to be offered patients) to patients’ life with a disease (Mol 2008). Contesting, however, an infrastructure that is fervently rooting for the optimization of precision medicine and, thus, creating multiple new options, can be harmful for care practices that largely depend on the virtuous attitude, character and human skills and sensitivity of the healthcare professionals. The differing demands posed by Mol’s two contrasting logics can be laborious to navigate and mitigate. Professional integrity can slowly erode when the logic of choice continuously outweighs the logic of care. Acknowledging that integrity matters in healthcare settings, as the Ethics Labs did, may in fact prove to be one way to mollify the tension between the logic of choice and the logic of care.

## The fertility of moral ambiquity

Moral ambiguity cannot be eliminated by resorting to pure materialism (the philosophical misstep of Cartesian dualism that to this day permeate our healthcare system), nor can it be resolved by escaping the sensible world in pure inwardness (Beauvoir 1948/2018, p. 8). The situated subject is not only rooted in materiality but interconnected with other situated subjects. Thus, subjects cannot be divorced from each other (Beauvoir 1948/2018, p. 72). It is through the (relational) engagement with other people that moral ambiguity can be conceptualized, clarified and negotiated. By acknowledging and discussing the ambiguous nature of the human condition, responsible, conscious moral action is, to Beauvoir, made possible. In contrast, by being unaware of the inherent ambiguity, moral decision-making may easily succumb to arbitrary, coincidental or habitual choices. Such a situation is undesirable in a field such as precision medicine with its vast ethical and societal implications.

As medicine and technology uncover the complex interplay between human phenotypes, genotypes and environmental exposures and develop precise targeting therapies, promising the individualization of patient care, moral ambiguity remains an underlying condition to be reckoned with. Yet, positioned within a rationalist tradition that prioritizes answers and solutions, “The culture of medicine evinces a deep-rooted unwillingness to acknowledge and embrace [uncertainty]” (Simpkin and Schwartzstein 2016, p. 1713). Unlike the common perception of moral ambiguity as being innately confusing, undesirable and something that ought to be immediately conquered, the Ethics Labs brought out a distinct fertile component. As demonstrated by the participants’ moral experiences, ambiguity can be considered a ubiquitous phenomenon in precision medicine. Creating a dialogical model such as the Ethics Laboratory that addresses doubts, dilemmas, concerns and obscurities within the field may not resolve them, nonetheless our study shows that it can foster moral literacy (Knox and Svendsen 2022). In this sense, moral ambiguity is not inherently bad, or as Beauvoir states: “Man must not attempt to dispel the ambiguity of his being but, on the contrary, accept the task of realizing it” (Beauvoir 1948/2018, p. 12). Facing ambiguity implies adopting an attitude of curiosity that can serve as an asset for precision medicine. This attitude does not intend to obliviate moral ambiguity but to acknowledge it. In this way, moral ambiguity injects humility (Reynolds 2018) into precision medicine, not in an attempt to denegrate its tremendous tools, for example diagnostic predictions that match diseases with particular biomarkers, but a humility about the scope of those tools. Instead of eliminating ambiguity, the participants get a chance to explore it. Reynolds defends the role of ambiguity in medical science and practice arguing that “to understand the practice of medicine as admitting of ambiguity is to admit that there are many cases where we don’t know, we can’t help, and even with the best laid plans, intentions, and science, we might simply be wrong” (Reynolds 2018, p. 3). This position was repeatedly aired in the Ethics Labs in various ways. One explained how predicting percentage for high risk of developing a genetic illness involves making, in his words, “an incredibly rough estimate” due to many unknown variables; another invoked the need to look at non-medical ways of knowing to access how patients and citizens understand care, well-being and the good life; yet another expressed the agony of not being able to help the vast majority of patients receiving personalized treatment and one recounted the discomfort of promising to protect personalized data in clinical trials without really knowing what happens to the data if analyzed abroad.

It is worth noting that it is not a specific situation, as such, that creates moral ambiguity. Moral ambiguity, as Beauvoir teaches us, is built into the world as a basic condition. A situation merely activates the awareness of it, yet this ambiguity is always in imminent risk of being overshadowed or deflected by busyness, routines and procedures. By deliberately examining the moral challenges of precision medicine, and in our case data, selection, interpretation and private–public partnerships, through the lens of moral ambiguity, the temptation of creating recipes for right action can be paused and participants can be allowed the time to increase moral sensitivity and exercise moral imagination. Yet, precision medicine traffics in a persuasive rhetoric of hope. In a recent article, Sandra Lee avers that “precision medicine has been steeped in promissory language of health, justice and human progress” (Lee 2020, p. 54). The danger is that this compelling language becomes so loud that the voice of confusion, caution and curiosity is stifled even among the professionals who are to develop, organize and implement precision medicine. As the Danish society, along with the global community, push for precision medicine, the politicians, clinicians and researchers are wise not to ignore the moral ambiguity it generates but, instead, allow space for it. By allowing it space in daily boundary work, moral ambiquity can become a fertile catalyst for ethical action. In addition, it can break open the philosophical sources of concepts such as normality, illness, health and well-being that are complex, changing and ambiguous.

In the performance-driven, competitive culture of precision medicine where science, industry and politics build a community of promise (Martin and Turkmenday 2021, p. 387), justice and integrity can seem secondary to promote. However, exploring moral ambiguity in its many variations, our research revealed how much justice and integrity are at the forefront of the participants’ consciousness. Both justice and integrity have long been argued for as foundational ethical principles in bioethics (Rendtorff 2002; Rendtorff and Kemp 2000) and, thus, protective guardrails to be acknowledged and respected. Feeling unprepared for the ethical implications of their work, as many of them were in their own mind, and therefore being at risk of compromising professional and/or personal values can constrain integrity and eventually deplete important moral resources and, as a consequence, expose oneself to the occurrence of moral distress (Hamric 2010; Epstein et al. 2019) and possibly burn-out. Ruminating on dense and thorny moral issues is a phenomenon that has been studied extensively among nurses but receiving increasing attention within other health care professions (Lamiani et al. 2017; Rasoal et al. 2017; Van den Bulcke et al. 2020). Due to its morally complex nature, we can expect similar studies on precision medicine in the future. Though there is no prefixed demarcation between right and wrong, good or bad, witnessing the participants’ genuine concern for holding the ethical line by advocating and acting with integrity and fairness may, in fact, be indicative of a moral vision that wishes to attune the goals of precision medicine with the irremediable ambiguities of reality.

## Footnotes

1 *Personalized Medicine in the Welfare State* (or MeInWe) is a social science project that was initiated in 2017 and runs until 2023. It examines the ethical, legal and organizational challenges in the implementation of precision medicine in Denmark. Project was made possible by Carlsberg Semper Arden grant with Mette Nordahl Svendsen as PI. Though we are aware that some have argued for differences between the term ‘personalized medicine’ and ‘precision medicine’, we use them interchangeably in this article.

2 We are alluding here to the ‘All of Us Research Program’ under the US NIH/National Institutes of Health that is intended to build a diverse health database.
